# Spontaneous complete necrosis of hepatocellular carcinoma: A case report and review of the literature

**DOI:** 10.3892/ol.2015.2937

**Published:** 2015-02-05

**Authors:** YUKI TAKEDA, NORITAKA WAKUI, YASUTSUGU ASAI, NOBUHIRO DAN, YOSHIYA YAMAUCHI, NOBUO UEKI, TAKAFUMI OTSUKA, NOBUYUKI OBA, SHUTA NISHINAKAGAWA, MASAMI MINAGAWA, YASUSHI TAKEDA, SAORI SHIONO, TATSUYA KOJIMA

**Affiliations:** 1 Department of Internal Gastroenterology and Hepatology, Tokyo Rosai Hospital, Tokyo 143-0013, Japan; 2 Department of Surgery, Tokyo Rosai Hospital, Tokyo 143-0013, Japan; 3 Department of Pathology, Tokyo Rosai Hospital, Tokyo 143-0013, Japan

**Keywords:** hepatocellular carcinoma, spontaneous necrosis, regression, fibrous capsule, arterio-portal shunt, thrombi

## Abstract

The present study reports the case of a 68-year-old male patient who presented to Tokyo Rosai Hospital for the treatment of alcoholic liver disease. A high density was observed in liver segment S2, while a tumor, 30 mm in size, exhibiting a low density was observed in the delayed phase upon contrast-enhanced computed tomography (CT), which was performed prior to admission. The tumor appeared slightly poorly defined upon abdominal ultrasound and was observed as a 30 mm low-echoic nodule that was internally heterogeneous. A 5-mm thick contrast enhancement effect was observed in the tumor border in the vascular phase on Sonazoid contrast-enhanced ultrasonography, while a defect in the entire tumor was observed in the post-vascular phase. Dysphagia had commenced three months prior to presentation and a weight loss of ~3 kg was observed. Therefore, the patient was admitted to Tokyo Rosai Hospital due to the presence of a hepatic tumor, and to undergo a close inspection of the cause of the tumor. Upon close inspection, it was determined that the weight loss and aphagia were caused by progressive bulbar paralysis. A contrast-enhanced CT was performed on post-admission day 29 as a follow-up regarding the hepatic tumor. As a result, although no change in the tumor size was observed, the contrast enhancement in the tumor borderline had disappeared. Necrosis of the tumor was considered. However, as viable persistence of the malignant tumor could not be excluded, a hepatic left lobe excision was performed. The patient was diagnosed with hepatocellular carcinoma (HCC) based on the morphology of the cellular necrosis. In addition, occlusion due to thrombus was observed within the blood vessels passing inside the fibrous capsule. It was hypothesized that the formation of a thick fibrous capsule and occlusion due to thrombus in the feeding vessel were possibly involved as the cause of complete spontaneous necrosis. Written informed consent was obtained from the patient.

## Introduction

Hepatocellular carcinomas (HCCs) are potentially life-threatening (1), whose prognosis is dependent on the tumor stage at the time of diagnosis and the possibility of providing radical treatment. Thanks to the development of clinically based staging systems such as the Barcelona Clinical Liver Cancer (BCLC) classification, which takes into account parameters as liver functionality (often as a consequence of underlying liver cirrhosis; Child-Pugh score), tumor burden (number and invasiveness), clinical performance and divides patients into very early/early, intermediate, advanced, and end-stage, the life expectancy of HCC patients can be reliably predicted and appropriate treatment may be identified (2). According to the classification system, some of the treatments including surgical resection, liver transplantation, percutaneous local radio frequency ablation, transarterial chemoembolization, palliative care are chosen (2). Recently the multikinase inhibitor sorafinib has been approved for use in patients with advanced HCC, for whom no therapy was previously available (3).

Spontaneous necrosis of a malignant tumor is rare, with an incidence of one case per 6,000–100,000 individuals (4). In cases with large HCC, spontaneous massive necrosis is common. It has been reported that cases of advanced necrosis that exhibit a necrosis rate of ≥90% within the HCC lesion account for ~2% of all HCCs (5). However, only 12 cases have been reported to not develop necrosis due to partial hepatectomy, or the pathological anatomy revealed that the patients did not possess HCC (1,6–16). Spontaneously necrotized HCC is considered to be a rare condition. Several mechanisms of spontaneous necrosis of HCC have been proposed, including a reduced blood supply to the cancer (such as capsule formation and arterial thrombosis), rapid tumor growth, immunological reaction (10), abstinence from drinking (17) and the use of herbal medicine (18). The present study reports the case of a patient with a spontaneously necrotized HCC, including its pathogenic mechanism.

## Case report

The present study reports the case of a 68-year-old male with alcoholic liver disease who was regularly treated at Tokyo Rosai Hospital (Tokyo, Japan). A 30 mm tumor was observed in the S2 liver segment upon abdominal contrast-enhanced computed tomography (CT), which was performed prior to admission. In addition, dysphagia had commenced three months prior and a weight loss of ~3 kg was observed. Therefore, the patient was admitted to Tokyo Rosai Hospital due to the presence of a hepatic tumor and in order to examine the cause of the tumor.

**Figure 1:**
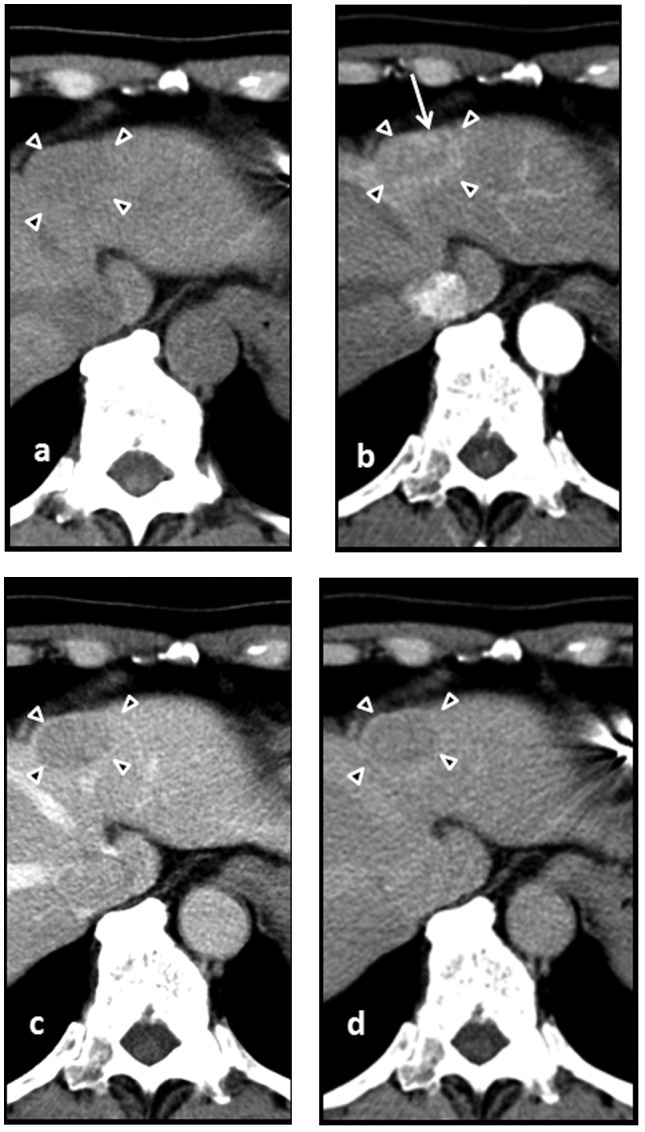
Contrast-enhanced computed tomography performed at the time of admission. A tumor 30 mm in size was observed in the S2 liver segment (arrowheads). A high density was indicated inside the tumor in the arterial phase (arrows), while a low density was indicated in the portal and delayed phases. (a) Plain; (b) arterial phase; (c) portal phase; (d) delayed phase.

**Figure 2:**
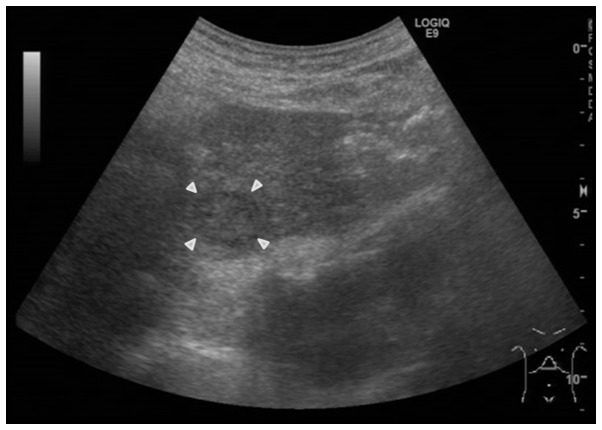
Abdominal ultrasound performed at the time of admission. A slightly poorly-defined high-echoic nodule 30 mm in size that was internally heterogeneous and comprised a low-echoic border was observed (arrowheads).

**Figure 3:**
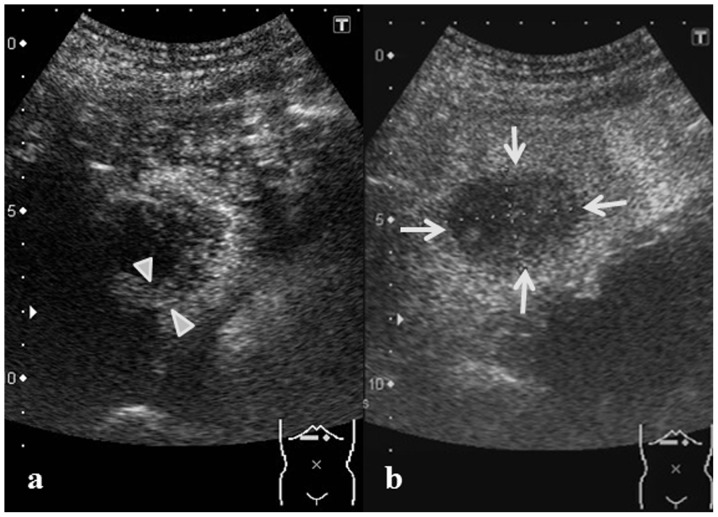
Abdominal contrast-enhanced ultrasonography at the time of admission. (a) A contrast enhancement effect of ~5 mm was indicated at the tumor border in the vascular phase in the tumor in the S2 liver segment (arrowheads). (b) A defect in the entire tumor was observed in the post-vascular phase (arrows).

The medical history of the patient included the implantation of a pacemaker, due to sick sinus syndrome, in 2011. The patient had consumed 1800 cc of Japanese sake per day (200 g ethanol/day) for 45 years, but had completely stopped this intake three years prior to the current presentation. The patient was 159 cm in height and weighed 48.3 kg, with a body mass index of 19.1, blood pressure of 109/69 mmHg, pulse rate of 79 beats/min and a body temperature of 37.3°C. Examination of the palpebral conjunctiva did not reveal anemia, and jaundice was not observed in the bulbar conjunctiva. Neither cardiac nor pulmonary murmurs were observed in the chest. The abdomen was flat and soft, with no pain upon the administration of pressure, and bowel sounds were normal. The liver and spleen were not palpated. There was no edema in the lower extremities and the superficial lymph nodes were not palpated. Spider angioma and palmar erythema were not observed. Upon blood testing, the blood platelet count was found to be 13.0×10^4^/μl (normal range, 14.0–37.9×10^4^/μl) and a low Na^+^ level of 133 mEq/l (normal range, 135–147 mEq/l) was identified, while the level of protein induced by vitamin K absence or antagonists II (PIVKA-II) was elevated, with a value of 427 milli-arbitrary units (mAu)/ml (normal level, <40 mAu/ml). No elevation in the levels of hepatic-cystic system enzymes or α-fetoprotein (AFP; 1.5 ng/ml; normal level, <10 ng/ml) were observed. No inflammatory or coagulating system abnormalities were observed, and no abnormalities were identified in the hepatitis B virus surface antigen (HBs-Ag) or hepatitis C virus antibody (HCV-Ab) levels (Table I). A tumor 30 mm in size was observed in the S2 liver segment upon abdominal contrast-enhanced CT, performed at the time of admission, which indicated a high density in the arterial phase and a low density in the portal and delayed phases (Fig. 1). Upon abdominal ultrasound, the tumor was found to be slightly poorly defined and was a 30 mm low echoic nodule that was internally heterogeneous (Fig. 2). A 5-mm thick region of enhancement was observed in the tumor border in the vascular phase upon Sonazoid contrast-enhanced ultrasonography. However, no enhancement was observed in the center of the lesion, while a defect in the entire tumor was observed in the post-vascular phase (Fig. 3).

Nutrition therapy was commenced immediately following admission by combining a dysphagia diet with tube feeding. Although a temporary inflammatory reaction and acceleration of the fibrinolytic system were identified on blood testing performed on post-admission day 8, with a D-dimer level of 1.9 mg/dl and a C-reactive protein (CRP) level of 8.1 mg/dl, these improved naturally. As a result of a subsequent in-depth inspection, the cause of dysphagia was identified as progressive bulbar paralysis. However, the bulbar paralysis was not considered to be associated with the carcinoma.

**Figure 4:**
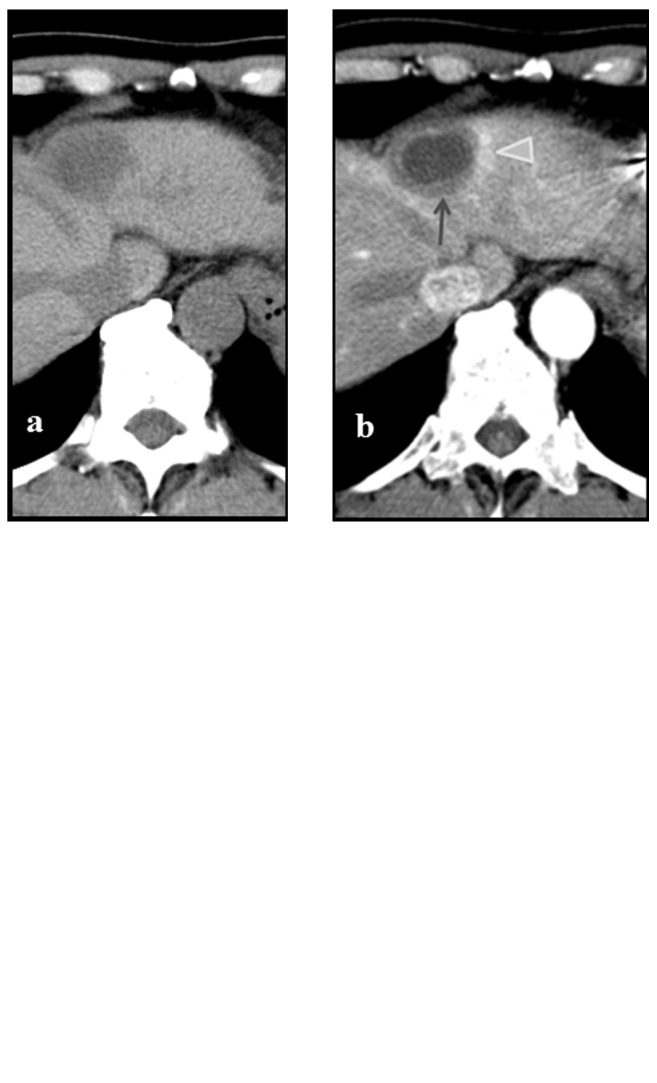
Contrast-enhanced computed tomography performed on post-admission day 29. The tumor diameter was ~30 mm in size, and did not exhibit internal contrast enhancement. However, a thick membrane of ~5 mm was gradually imaged in the vicinity of the tumor (arrow), and a deeply-stained region with a 5 mm diameter, considered to be an arterio-portal shunt, was observed in the vicinity of this region (arrowhead). (a) Plain; (b) arterial phase; (c) portal phase; (d) delayed phase.

**Figure 5:**
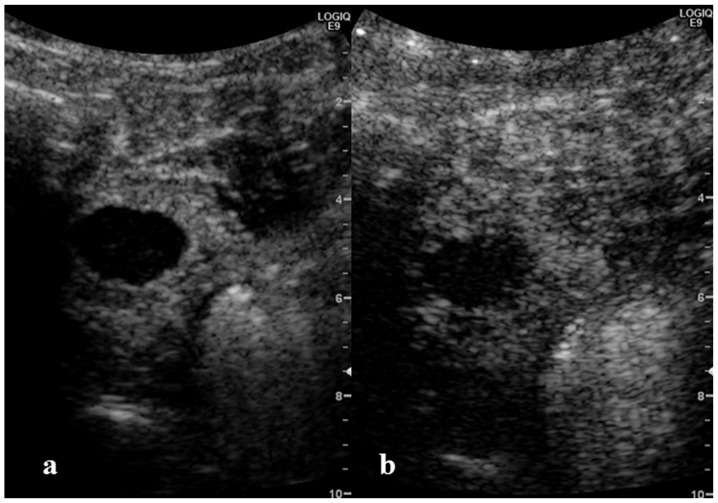
Abdominal contrast-enhanced ultrasonography 30 days post-admission. No stained image was observed in the (a) vascular and (b) post-vascular phases, and a defect of a~30 mm was observed.

The AFP level increased slightly to 1.8 ng/ml and the PIVKA-II decreased by 41.0 mAU/ml, as observed on blood testing on post-admission day 23. An additional abdominal contrast-enhanced CT was performed on post-admission day 29, revealing no change in the tumor size, which remained at ~30 mm. However, the contrast enhancement of the tumor observed at the time of admission was no longer present, and a faint contrast enhancement that was 5 mm in width was instead observed surrounding the tumor. Additionally, a 5-mm wide deeply-stained region, hypothesized to be an arterio-portal (A-P) shunt was observed in the vicinity of the tumor (Fig. 4). The vascular and post-vascular phases of Sonazoid contrast-enhanced ultrasonography (Sonazoid perfluorobutane; GE Healthcare, Oslo, Norway), performed on post-admission day 30, revealed no enhancement in the tumor, but a defect ~30 mm in size was observed (Fig. 5). Necrosis of the tumor was suspected. However, as viable persistence of the malignant tumor could not be excluded, a hepatic left lobe excision was performed on post-admission day 43.

**Figure 6:**
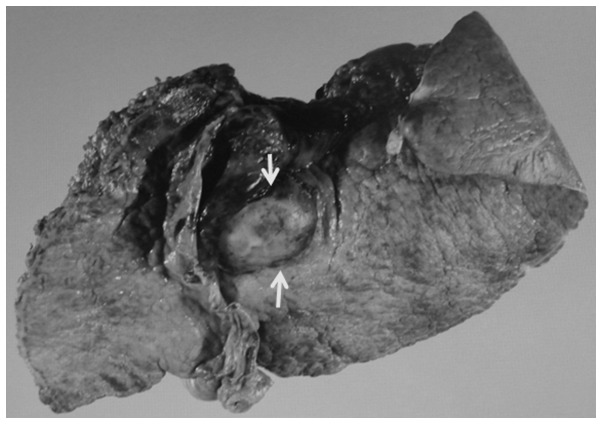
Image of the excised specimen. A 25×18 mm well-defined white-colored circular tumor was observed in the S2 liver segment (arrow).

A visual inspection of the excised specimen revealed a 25×18 mm well-defined white-colored circular tumor in the S2 liver segment (Fig. 6).

**Figure 7:**
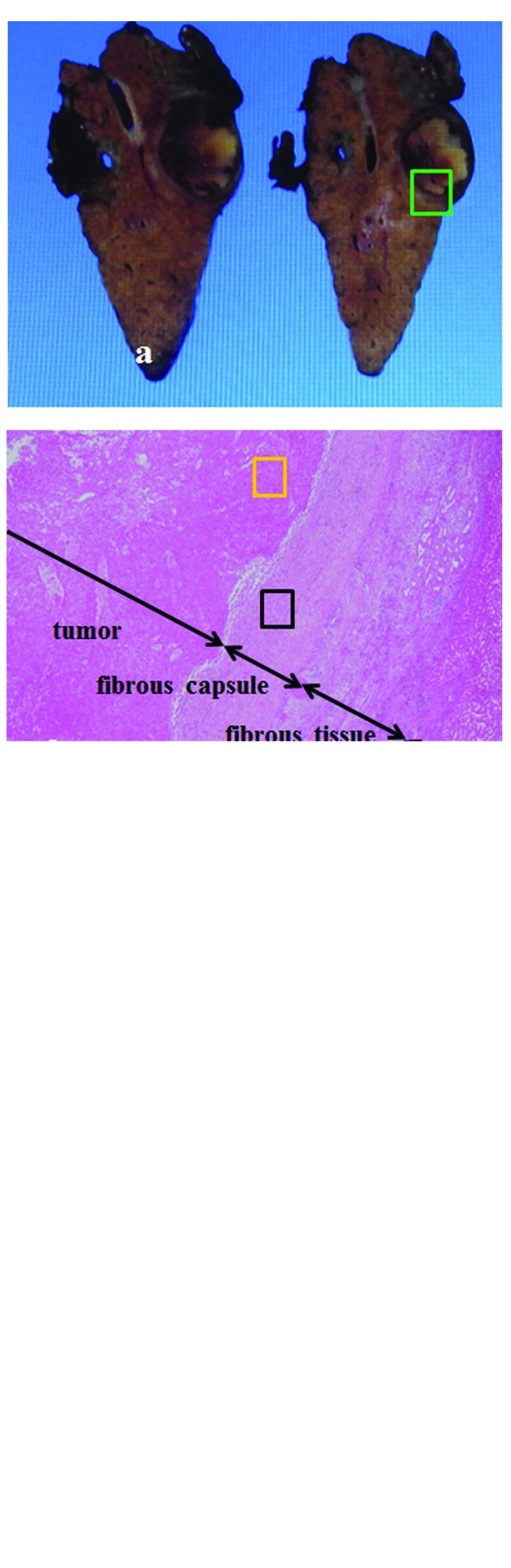
Histopathological image of hepatocellular carcinoma tissue. (a) Macroscopic findings: Two consecutive sections. (b) Magnification of the green-bordered section of (a) (HE stain; magnification, ×40). The formation of a~1-mm thick fibrous capsule surrounding the tumor was observed, and the entire region within the tumor was necrotized. Outside the fibrous capsule, fibrous tissue ~3 mm thick was observed. The background liver disease was chronic hepatitis. (c) Magnification of the yellow-bordered section of (b) (HE stain; magnification, ×40). Cellular necrosis is considered to be a valvate-arrangement of oval cells, which was observed within the tumor. (d) Magnification of the black-bordered section of (b) (HE stain; magnification, ×100). Occlusion due to thrombus was observed within the blood vessels passing inside the fibrous capsule (arrowheads).

Pathological imaging revealed the formation of a fibrous capsule ~1 mm in size surrounding the tumor, and the entire region within the tumor was necrotized. As cellular necrosis is considered to be a valvate-arrangement of oval cells, which was observed in a region of the present tumor, the resulting diagnosis was HCC in which necrosis had commenced. Outside the fibrous capsule, ~3 mm of fibrous tissue was observed. Occlusion due to thrombus was observed within the blood vessels passing through the fibrous capsule. The background liver disease was chronic hepatitis (Fig. 7).

Eight months subsequent to admission, no re-increase of the tumor marker was observed and no recurrence of HCC was identified in the follow-up CT.

## Discussion

Although a pre-operative histological diagnosis was not performed for the present patient, HCC was suspected due to the presence of alcoholic liver disease, which was the causative factor of HCC, an increased PIVKA-II value and the tumor exhibiting a high density in the arterial phase and a low density in the portal and delayed phases of abdominal contrast-enhanced CT, which was performed at the time of admission. The possibility of a metastatic hepatic tumor and cholangiocarcinoma was considered. However, findings that indicated the presence of a malignancy other than a hepatic tumor were not observed upon close systematic inspection, and as no expansion of the intrahepatic bile duct was observed in the vicinity of the tumor, the possibility of a different malignancy was considered to be low. The contrast enhancement at the tumor border observed upon contrast-enhanced CT at admission was not observed at all on the CT performed on the post-admission day 29 and necrosis within the tumor was suspected. However, the persistence of cancer could not be ruled out. As a result, and due to the cellular necrosis being considered to be a valvate-arrangement of oval cells, which was observed within the fibrous capsule, a diagnosis of HCC in which necrosis was histopathologically caused was made. In addition, the entire tumor was necrotized.

The possible causes of spontaneous necrosis of HCC include hepatic circulation impairment due to massive bleeding or shock (6,19), damage to the lining fibrous capsule of the artery supplying the tumor due to catheterization (20), a sudden enlargement of the tumor (7), a reduced blood supply to the cancer nodule due to the formation of a fibrous capsule (7), damage to the cancer nodule due to inflammatory cells, such as lymphocytes, and cytokines, such as TNF-α (7,9,21), abstinence from drinking (18), and the use of herbal medicines (18). It has been reported that HCCs are generally observed with various levels of necrosis depending on the post-operative histopathology (5).

HCC, in which complete necrosis is histopathologically diagnosed is rare. A search of the literature on PubMed (National Center for Biotechnology Information, US National Library of Medicine, Bethesda, MD, USA) was performed using the keywords ‘spontaneous complete necrosis of hepatocellular carcinoma AND regression’, and 12 cases published between 1978 and 2012 were found, in addition to the present case (Table II). Not all studies are included in the table, as the results contained cases diagnosed by only diagnostic imaging. Upon investigating the reported cases, an increased number of male patients was found compared with female patients, namely 11 cases, and the mean age was 67 years old. The smallest tumor was 30 mm in size and the largest was 130 mm in size, with a mean of 67 mm. The cause of hepatic disease was HBV in four cases, HCV in three cases and alcohol in two cases, including overlapping cases without an apparent tendency, and the background hepatic disorder was chronic hepatitis in five cases and liver cirrhosis in four cases, exhibiting no apparent bias. The cause of the spontaneous necrosis of the tumor was, including overlapping cases, reported to be the formation of a fibrous capsule in 10 cases, immunological reaction in eight cases, artery occlusion in five cases, portal vein embolization in one case, massive bleeding in one case and herbal medicine in one case, with no apparent tendency, although many cases exhibited overlapping symptoms.

In the present study, occlusion of the artery supplying the carcinoma, due to the formation of thrombus, and the formation of a fibrous capsule were observed, causing declined blood flow to the tumor. This resulted in rapid complete spontaneous necrosis of the tumor. Although the cause of the thrombus formation is unknown, the present patient exhibited a dehydrating tendency due to dysphagia resulting from progressive bulbar paralysis, and was in a state prone to the formation of thrombus due to undergoing pacemaker implantation. Overall, these factors were surmised to be the causes of the hepatic tumor. A temporary inflammatory reaction was observed on post-admission day 8, indicated by a CRP level of 8.1 mg/dl, and it was hypothesized that necrosis of the tumor progressed over the same period.

Although no uniform conclusion has been obtained regarding the treatment of cases with complete spontaneous necrosis of HCC, relapse of spontaneously necrotized HCC has been reported (22) and viable persistence of the tumor cells has also been often observed, despite advanced necrosis being diagnosed in imaging examinations (5). It is hypothesized that the same treatment should be administered to patients with advanced necrosis as is administered to those with general HCC, while taking into consideration the possibility of persisting tumor cells.

In the present study, a patient with HCC that developed spontaneous complete necrosis was admitted to the Tokyo Rosai Hospital, with the diagnosis being confirmed by surgical resection and pathological evaluation. The cause of the complete spontaneous necrosis was surmised to be occlusion of the blood vessel supplying the tumor, due to the formation of thrombus, along with the formation of a thick fibrous capsule and large ischemia generated in the tumor. This phenomenon provides a valuable insight into spontaneous complete necrosis of HCC.

## Figures and Tables

**Table I:** Blood laboratory findings on admission.

Diagnostic blood tests	Result
Biochemistry	
CRP	0.2 mg/dl
Na	133 mEq/l
K	4,1 mEq/l
Cl	93 mEq/l
TP	7.3 g/dl
Alb	4.2 g/dl
T-Bil	0.5 mg/dl
AST	30 IU/l
ALT	38 IU/l
LDH	139 IU/l
ALP	243 IU/l
GGT	50 IU/l
LDL-C	78 mg/dl
HDL-C	56 mg/dl
TG	70 mg/dl
BUN	8 mg/dl
Cr	0.73 mg/dl
PT	88%
PT-INR	1.07
Hematology	
WBC	6000/μl
RBC	421×10^4^/μl
Hgb	14.4 g/dl
Hct	41.3%
PLT	13.0×10^4^/μl
Serology	
HCV-Ab	0.1 COI
HBs-Ag	0.02 IU/l
HBc-Ab	-
ANA	40
AMAM2	<0.5
Tumor markers	
CEA	1.5 ng/ml
CA19-9	2 U/ml
AFP	1.5 ng/ml
PIVKA II	427 mAU/ml

CRP, C-reactive protein; Na^+^, sodium; K^+^, potassium; Cl^−^, chlorine; TP, total protein; Alb, albumin; T-Bil, total bilirubin; AST, aspartate aminotransferase; ALT, alanine aminotransferase; LDH, lactate dehydrogenase; ALP, alkaline phosphatase; GGT, gamma-glutamyltranspeptidase; LDL-C, low density lipoprotein-cholesterol; HDL-C, high density lipoprotein-cholesterol; TG, triglyceride; BUN, blood urea nitrogen; Cr, creatinine; PT, prothrombin time; INR, international normalized ratio; WBC, white blood cell count; RBC, red blood cell count; Hgb, hemoglobin; Hct, hematocrit; PLT, platelet count; HCV-Ab, hepatitis C antibody; HBs-Ag, hepatitis B antigen; HBc-Ab, hepatitis B antibody; ANA, anti-nuclear antibody; AMAM2, anti-mitochondrial M2 antibody; CEA, carcinoembryonic antigen; CA, carbohydrate antigen; AFP, α-fetoprotein; PIVKA II, protein induced by vitamin K absence or antagonists II; mEq; milliequivalents; IU, international unit; COI, cutoff index; U, units; mAU, milli-arbitrary units.

**Table II:** Analysis of the reported patients with hepatocellular carcinoma undergoing spontaneous complete necrosis.

Study	Author, Year (ref)	Patient age	Gender	HBV or HCV	Alcoholic liver disease	Tumor size, mm	Capsule formation	Liver disease	Factors involved in necrosis
1	Our case, 2012	68	M	No	Positive	30	Positive	CH	A-P shunt, thrombi
2	Yokoyama *et al*, 2012 (11)	80	M	No	ND	68	Positive	ND	Imune, thrombi
3	Maejima *et al*, 2011 (10)	68	M	HCV	Positive	100	Positive	CH	Imune
4	Arakawa *et al*, 2008 (12)	78	F	HBV	No	30	Positive	ND	Imune
5	Ohta *et al*, 2005 (1)	74	M	No	ND	60	Positive	ND	Imune, thrombi
6	Li *et al*, 2003 (13)	53	M	HBV	No	30	ND	LC	Imune
7	Iiai *et al*, 2003 (14)	69	M	HCV	ND	40	Positive	CH	Portal vein thrombi
8	Morimoto *et al*, 2002 (15)	73	M	No	Positive	100	Positive	CH	Thrombi
9	Izuishi *et al*, 2000 (7)	50	M	HCV	ND	40	Positive	CH	Imune
10	Ozeki *et al*, 1996 (16)	69	F	ND	No	30	Positive	LC	Imune
11	Markovic *et al*, 1996 (9)	62	M	HBV	No	130	Positive	LC	Imune
12	Gaffy *et al*, 1990 (6)	63	M	No	No	100	ND	LC	Herbs, bleeding
13	Andreola *et al*, 1987 (8)	75	M	HBV	ND	120	Positive	ND	Thrombi

M, male; F, female; HBV, hepatitis B virus; HCV, hepatitis C virus; LC, liver cirrhosis; CH, chronic hepatitis; ND, not described; A-P shunt, arterio-portal shunt; Imune, immunological reaction.

## Abbreviations

HCC: hepatocellular carcinoma
CT: computed tomography
PIVKA- II: Protein induced by vitamin K absence or antagonists II
A-P: arterio-portal
